# Cellular Changes in Blood Indicate Severe Respiratory Disease during Influenza Infections in Mice

**DOI:** 10.1371/journal.pone.0103149

**Published:** 2014-07-24

**Authors:** Leonie Dengler, Nora Kühn, Dai-Lun Shin, Bastian Hatesuer, Klaus Schughart, Esther Wilk

**Affiliations:** 1 Department of Infection Genetics, Helmholtz Centre for Infection Research, Braunschweig, Germany; 2 University of Veterinary Medicine Hannover, Hannover, Germany; 3 University of Tennessee Health Science Center, Memphis, Tennessee, United States of America; Washington University School of Medicine, United States of America

## Abstract

Influenza A infection is a serious threat to human and animal health. Many of the biological mechanisms of the host-pathogen-interactions are still not well understood and reliable biomarkers indicating the course of the disease are missing. The mouse is a valuable model system enabling us to study the local inflammatory host response and the influence on blood parameters under controlled circumstances. Here, we compared the lung and peripheral changes after PR8 (H1N1) influenza A virus infection in C57BL/6J and DBA/2J mice using virus variants of different pathogenicity resulting in non-lethal and lethal disease. We monitored hematological and immunological parameters revealing that the granulocyte to lymphocyte ratio in the blood represents an early indicator of severe disease progression already two days after influenza A infection in mice. These findings might be relevant to optimize early diagnostic options of severe influenza disease and to monitor successful therapeutic treatment in humans.

## Introduction

Each year the influenza A virus infects approximately 500 million people worldwide, of which about 500,000 die [1]. In recent history, the emergence of new influenza subtypes has caused severe pandemics [2], [3]. The pandemic of 1918, the most severe to date, led to 30–50 million deaths worldwide [4]. A new variant of a seasonal H1N1 virus, pH1N1, caused a worldwide pandemic in 2009 [5], [6]. In addition, avian viruses, such as H7N9 and H5N1, are also able to infect humans with high mortality rates [7], [8], [9]. In humans it is very difficult to predict the course and severity of an influenza infection in the lung and to monitor the efficacy of therapies [10], [11]. Currently, blood analysis is performed which mainly consists of measurements of general inflammatory markers that do not allow evaluating the actual status of the disease. IL6 has been suggested as a potential biomarker for severe H1N1 infections in humans [12]. One study has so far been performed in humans in which blood transcriptome analysis was implemented in experimentally infected human volunteers [13].

A large amount of information has been accumulated from experimental animal studies [14], [15], [16], [17], [18], [19]. However, these data were very seldom correlated with biomarkers in body fluids. Thus, there is an urgent need for reliable biomarkers to identify parameters for early prognosis of severe disease progression in humans. The use of systematic experimentally well controlled studies in animal models will represent an important step forward in this respect.

Here, we investigated the host response of C57BL/6J and DBA/2J mice to infections with PR8 (H1N1) virus variants displaying different pathogenicity. Our results indicate that a severe course of the disease is reflected early after infection in several peripheral parameters. Especially, the granulocyte to lymphocyte ratio could be identified as indicative parameter for the disease severity already on day two after infection.

## Results

### Lethal and non-lethal murine infection models revealed significant differences in hematological parameters

To compare the host response in infection models of different severity, we used two variants of PR8 virus with different pathogenicity, PR8M and PR8F. These two PR8 variants were derived from the same isolate but have different passage histories and were obtained from different laboratories (PR8M in Muenster [20] and PR8F in Freiburg [21]). Two inbred mouse strains, C57BL/6J and DBA/2J, with known differences in their susceptibility were infected with these viruses. Infection of C57BL/6J mice with 2×10^3^ FFU (focus forming units) PR8F virus resulted in continuous body weight loss and all animals succumbed to the infection on day five to six post infection (p.i.) (Figure IA.b in File S1). Viral loads in PR8F infected lungs reached a level of about 100-fold higher compared to PR8M infected mice (IA.a in File S1). Histological analyses revealed a massive and more pronounced myeloid cell infiltration already by day 2 p.i. in PR8F (Figure IB.h in File S1) compared to PR8M infected mice (Figure IB.b in File S1). Damage to lung epithelial cells was also more advanced after infection with PR8F virus (Figure IB.h, i and b, c in File S1). Furthermore, oxygen saturation in the blood decreased already by day 3 p.i. in the lethal model (PR8F), indicating the early impact of the infection process on lung function (Figure IA.b in File S1).

After infection with 2×10^3^ FFU of the non-lethal PR8M, C57BL/6J mice survived the infection (Figure IA.a in File S1). Oxygen saturation decreased steadily from day 5 to day 8 p.i. in parallel with the expanding infiltration of lymphocytic cells (Figure IA.a, IB.d, e in File S1). Our results confirm previously published observations of a direct correlation between oxygen saturation and lung pathology after PR8 infection in mice and its impact on disease outcome [22]. In addition, we investigated the host response in DBA/2J mice that are highly susceptibility to influenza A infections. An infection with 2×10^3^ FFU PR8M virus results in a lethal outcome on days 6 to 7 p.i. (Figure IA.c in File S1 and [17], [21]).

**Figure 1:**
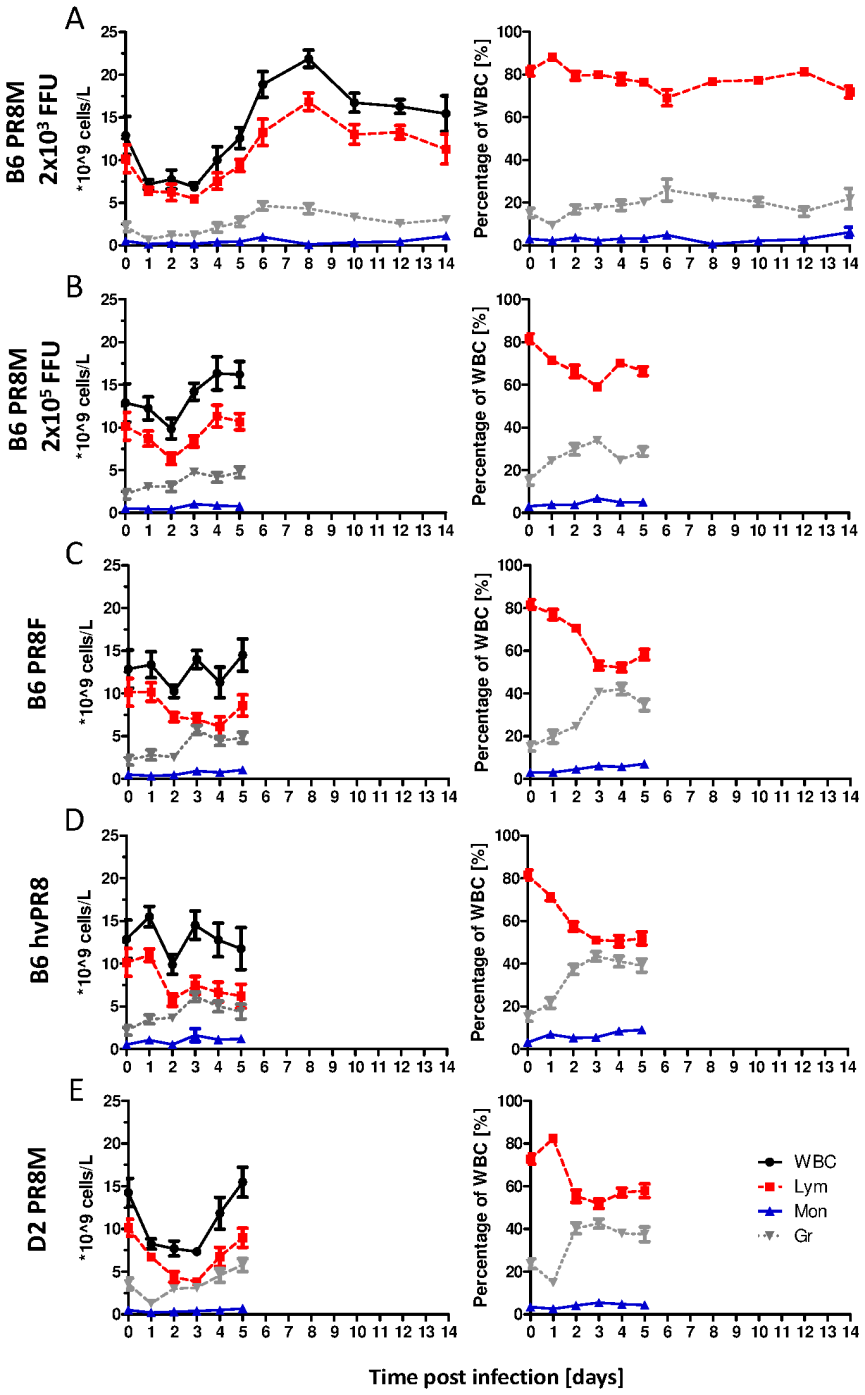
Hematological parameters in the blood exhibited distinct differences in C57BL/6J & DBA/2J mice infected with different PR8 virus variants. The left graphs depict the kinetics of absolute numbers of white blood cells (WBC), lymphocytes (Lym), granulocytes (Gr) and monocytes (Mon); the right panel illustrates the relative amount of lymphocytes, monocytes and granulocytes: C57BL/6J mice infected with 2×10^3^ FFU PR8M (A); 2×10^5^ FFU PR8M (B); 2×10^3^ FFU PR8F (C); 2×10^3^ FFU hvPR8 (D) and DBA/2J infected with 2×10^3^ FFU PR8M (E). Results are presented as mean±SEM and are based on two independent experiments for the first five days p.i. (n = 10) and from one experiment for days 6–14 (n = 5).

To detect changes in the periphery, we generated hemograms of the blood (Figure 1, Tables I-V in File S1). Non-lethal infections in C57BL/6J infected with 2×10^3^ PR8M induced a lymphopenia during the first three days p.i. followed by an increase of absolute numbers of lymphocytes and total white blood cells (WBC) (Figure 1A), resulting in a leukocytosis on days 6 to 8. Only a slight increase in neutrophils and monocytes was observed until day 6 p.i. Infections of C57BL/6J mice with a higher dose of PR8M (2×10^5^ FFU) revealed increased severity but still 100% of mice survived. The change of hematological parameters (Figure 1B and Table II in File S1) showed a further increase of granulocytes compared to C57BL/6J mice infected with the lower dose. During the lethal infection of C57BL/6J with PR8F a steady decrease of lymphocytes and a concomitant increase of granulocytes were observed (Figure 1C). In addition, the absolute amount of monocytes was five times higher in blood of PR8F versus PR8M infected mice on day 3 p.i. (Figure 1A+C, Tables I+III in File S1). Kinetics of absolute leukocyte counts in PR8M infected DBA/2J and C57BL/6J mice were similar, but DBA/2J mice exhibited a strong lymphopenia, combined with a granulocytosis (Figure 1E, Table V in File S1). To further evaluate the influence of influenza A virus pathogenicity on the hemogram, we infected mice with a highly virulent variant of PR8 (hvPR8) [23] (Figure 1D and Table IV in File S1).

### Granulocyte to lymphocyte ratio indicates the severity of the infection

**Figure 2:**
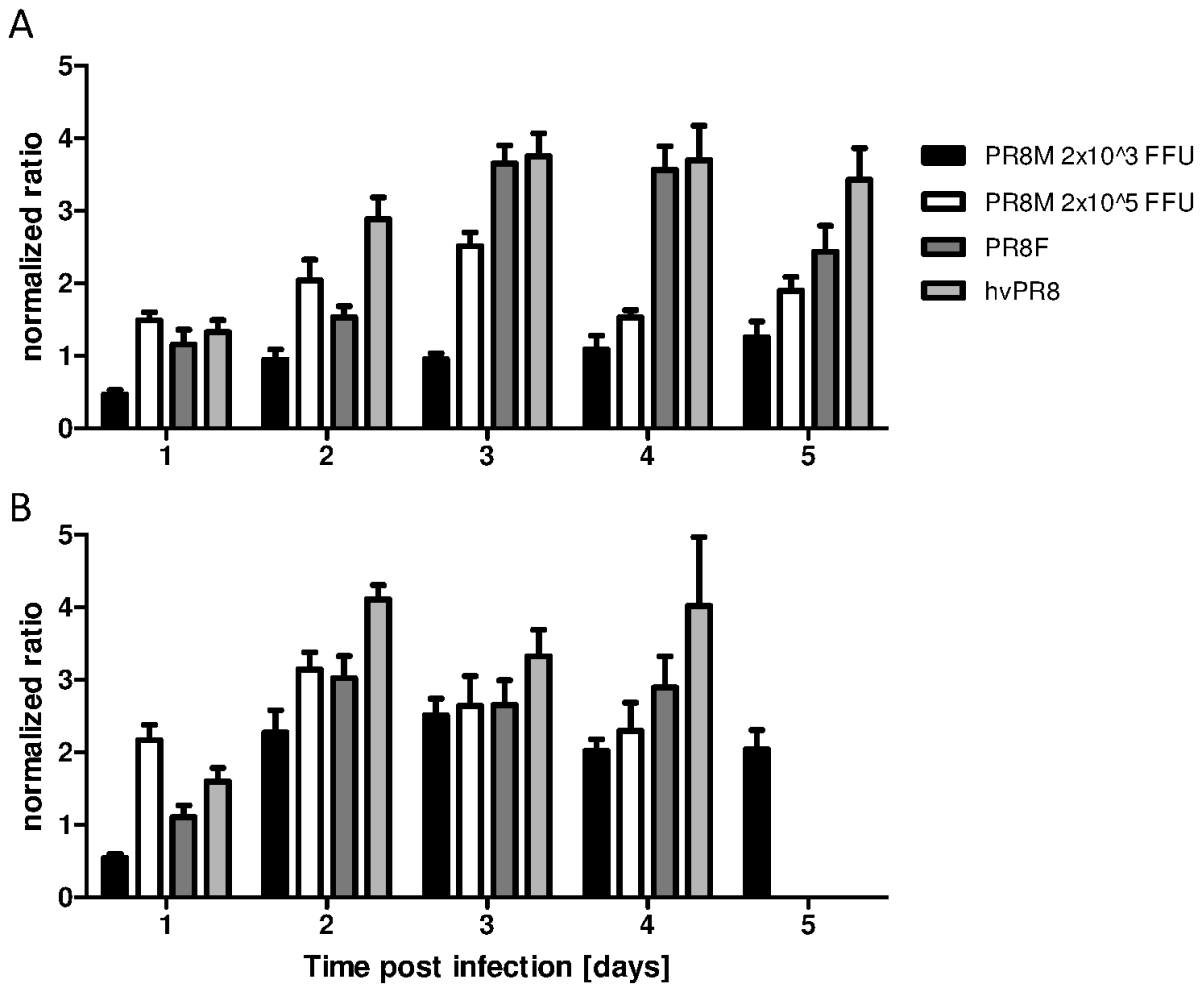
Normalized ratio of granulocytes to lymphocytes increased in dependence of disease severity in C57BL/6J & DBA/2J mice after influenza A infection. The normalized ratio of granulocytes to lymphocytes (ratio(dx) = ([granulocytes(dx)]/[lymphocytes(dx)])/ratio(d0)) was calculated from absolute cell counts. C57BL/6J (A) and DBA/2J mice (B) were infected with three variants of PR8 with a dose of 2×10^3^ FFU and in addition with a higher dose of the low pathogenic PR8M virus (2×10^5^ FFU). Results are presented as mean±SEM and are based on two independent experiments for the first five days p.i. (n = 10). Statistical analysis is presented in Tables 1 and 2.

Based on the hematological data described above, we calculated the ratio of granulocytes to lymphocytes during the course of infection (Figure 2). The granulocyte to lymphocyte ratio correlated well with the severity of the infection. Whereas only a slight increase over time in this ratio was observed in low dose PR8M infected C57BL/6J, a higher ratio was already evident on day 1 p.i. in high dose PR8M and lethally infected C57BL/6J mice (Figure 2A). Comparing the non-lethal high dose PR8M with the lethal PR8F infection, significant differences were obtained on days 3 and 4 indicating that the kinetics of this parameter are related to disease severity (Figure 2A and Table 1). In the case of infections with hvPR8, the increase of the granulocyte to lymphocyte ratio was even more pronounced than for lethal PR8F infections revealing a significant difference between PR8F and hvPR8 infections on day 2 p.i. (Figure 2A and Table 1).

**Table 1:** Pair wise statistical comparison of all granulocyte to lymphocyte ratios of C57BL/6J presented in Figure 2A.

		PR8M	PR8M	PR8F
		2×10^3^ FFU	2×10^5^ FFU	2×10^3^ FFU
Day 1	PR8M 2×10^5^ FFU	****		
	PR8F 2×10^3^ FFU	*	n.s.	
	hvPR8 2×10^3^ FFU	**	n.s.	n.s.
Day 2	PR8M 2×10^5^ FFU	**		
	PR8F 2×10^3^ FFU	*	n.s.	
	hvPR8 2×10^3^ FFU	****	*	***
Day 3	PR8M 2×10^5^ FFU	****		
	PR8F 2×10^3^ FFU	***	**	
	hvPR8 2×10^3^ FFU	****	**	n.s.
Day 4	PR8M 2×10^5^ FFU	n.s.		
	PR8F 2×10^3^ FFU	***	****	
	hvPR8 2×10^3^ FFU	****	****	n.s.
Day 5	PR8M 2×10^5^ FFU	*		

Data were analyzed for statistically significant differences using non-parametric Mann-Whitney-U-test. *: p-value<0.05; **: p-value<0.01; ***: p-value<0.001; ****: p-value<0.0001.

In addition DBA/2J mice were infected with the same viruses and doses. All infection protocols were lethal for the highly susceptible DBA/2J mice, but revealed significant differences of the granulocyte to lymphocyte ratio correlating with the pathogenicity of the applied virus (Figure 2B and Table 2). The higher virulence of PR8F and hvPR8 was still inducing even higher granulocyte to lymphocyte ratios (Figure 2B). DBA/2J versus C57BL/6J infected with 2×10^3^ PR8M (low dose) revealed a much more pronounced increase of the ratio starting from day 2 p.i. Thus, the severity of the infection was also reflected in the hemogram if the fatal outcome was due to increased genetic susceptibility of the host. To verify the validity that the granulocyte to lymphocyte ratio indicates disease severity, we calculated the correlation of the ratio and viral load for seven different infection experiments (C57BL/6J: PR8M 2×10^3^ FFU and 2×10^5^ FFU, PR8F 2×10^3^ FFU, hvPR8 2×10^3^ FFU; DBA/2J: PR8M, PR8F, hvPR8 all 2×10^3^ FFU; data from Figure I in File S1 and [21]). Correlation analysis revealed significant values on all days tested starting already on day 1 after infection (Table 3).

**Table 2:** Pair wise statistical comparison of all granulocyte to lymphocyte ratios of DBA/2J presented in Figure 2B.

		PR8M	PR8M	PR8F
		2×10^3^ FFU	2×10^5^ FFU	2×10^3^ FFU
Day 1	PR8M 2×10^5^ FFU	****		
	PR8F 2×10^3^ FFU	*	**	
	hvPR8 2×10^3^ FFU	****	n.s.	*
Day 2	PR8M 2×10^5^ FFU	*		
	PR8F 2×10^3^ FFU	n.s.	n.s.	
	hvPR8 2×10^3^ FFU	***	**	**

Data were analyzed for statistically significant differences using non-parametric Mann-Whitney-U-test. *: p-value<0.05; **: p-value<0.01; ***: p-value<0.001; ****: p-value<0.0001.

**Table 3:** Nonparametric Spearman correlation coefficient was calculated for viral load in the lung and granulocyte to lymphocyte ratio in the blood using data sets from seven infection models: C57BL/6J: PR8M 2×10^3^ FFU and 2×10^5^ FFU, PR8F 2×10^3^ FFU, hvPR8 2×10^3^ FFU; DBA/2J: PR8M, PR8F, hvPR8 all 2×10^3^ FFU (see Figure I in File S1 and [21]).

	Day 1	Day 2	Day 3	Day 4
Number of XY Pairs	7	7	7	7
r_s_	0,8571	0,8214	0,8929	0,7857
P value	0,0238	0,0341	0,0123	0,0480

A more detailed investigation of the blood from PR8M (low dose) and PR8F infected C57BL/6J mice by flow cytometry confirmed the higher levels of granulocytes and monocytes in C57BL/6J mice after PR8F infection obtained in the hematological study (data not shown). Also, the analysis of the lymphocyte population revealed a significantly higher proportion of CD8^+^ T cells in PR8F infected mice (Figure II in File S1).

### Red blood cell and platelet parameters were not associated with disease severity

**Figure 3:**
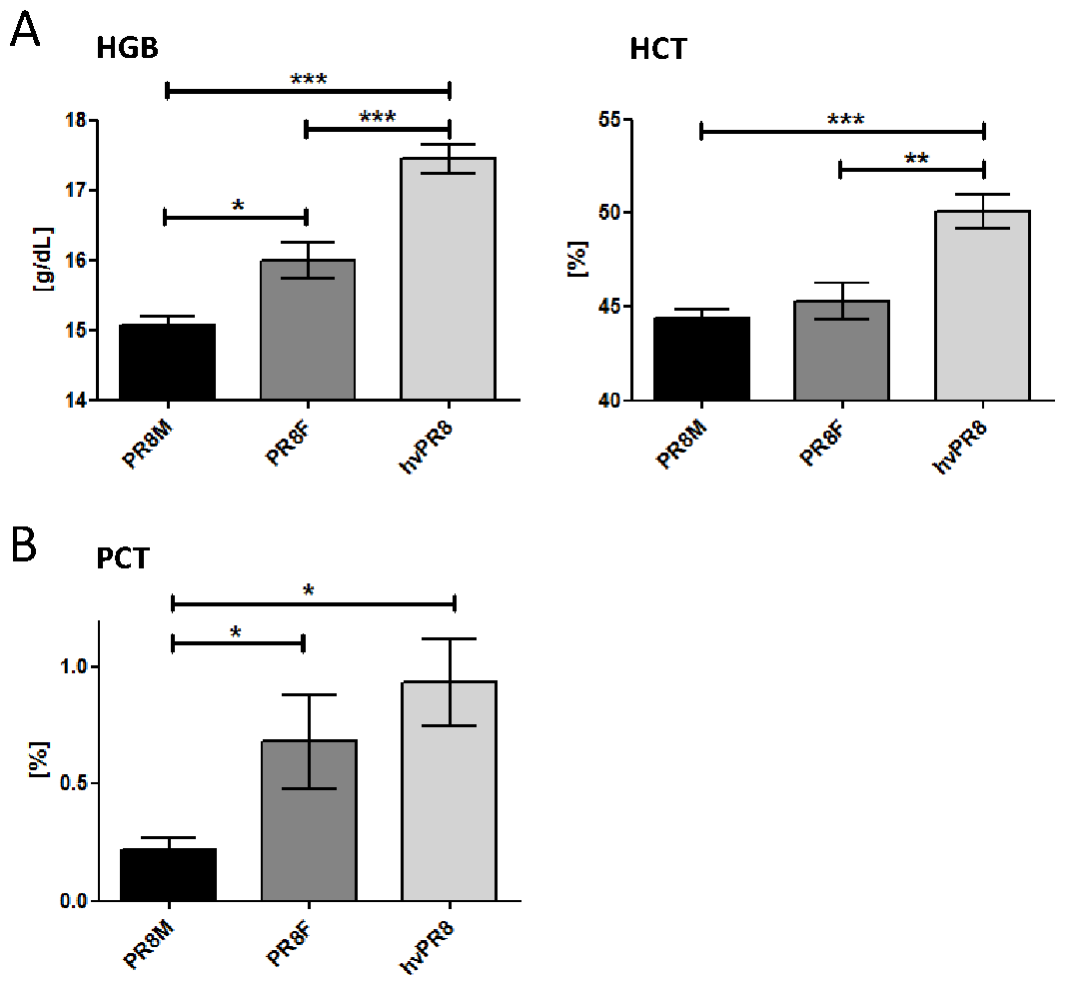
Red blood cell and platelet related parameters after IAV infection. Two erythrocyte parameters, hemoglobin (HGB), hematocrit (HCT), were significantly different between PR8M, PR8F and hvPR8 infected C57BL/6J mice (2×10^3^FFU) on day 5 after infection (A). Also changes in the plateletcrit (PCT) values were significantly different between PR8M, PR8F and hvPR8 infected C57BL/6J mice (2×10^3^ FFU) on day 5 (B). Data were collected from two independent experiments (n = 10) for the first five days p.i. Data were analyzed for statistically significant differences using non-parametric Mann-Whitney-U-test. *: p-value<0.05; **: p-value<0.01; ***: p-value<0.001.

In addition to WBC, also red blood cell and platelet-related parameters were investigated (Figure 3 and Figure III + IV in File S1). The comparison of C57BL/6J infected with PR8M (low dose), PR8F and hvPR8 revealed significant differences (Figure 3). The changes in hemoglobin (HGB) and hematocrit (HCT) were significantly different between lethally and non-lethally infected mice on day 5 p.i. corresponding to the virulence of the virus (Figure 3A). Furthermore, the increase of plateletcrit (PCT) correlated positively with the severity of infection on day 5 p.i. (Figure 3B). However, these associations were limited to data derived from C57BL/6J and were not found in infected DBA/2J mice (data not shown).

### C57BL/6J mice infected with lethal or non-lethal variants of PR8 revealed quantitative and qualitative differences in immune cell infiltrates of the lung

**Figure 4:**
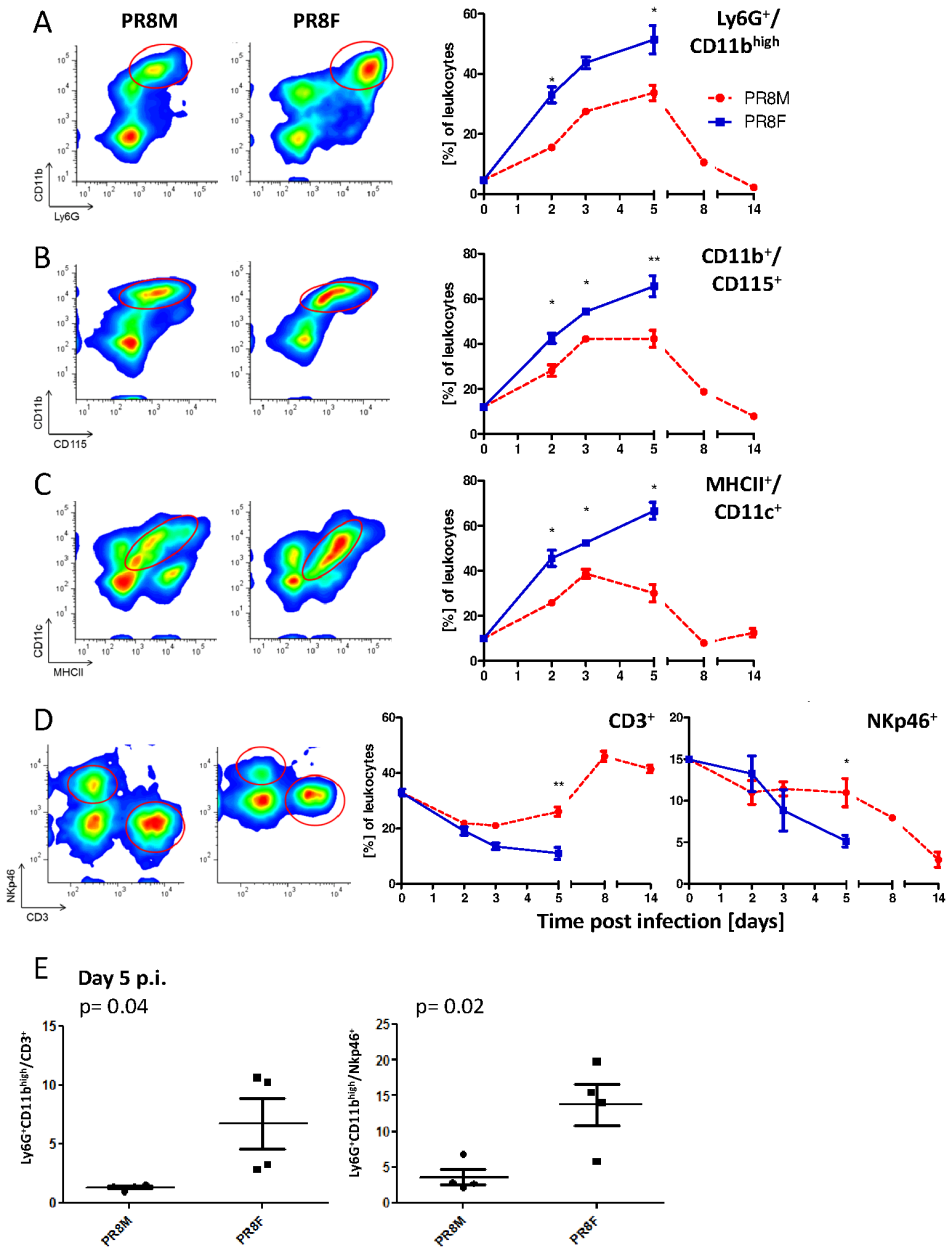
Flow cytometric analyses revealed quantitative and qualitative differences of the host response to PR8M versus PR8F infection. Lungs from infected C57BL/6J mice (2×10^3^ FFU) were extracted and cell suspensions were analyzed by flow cytometry on days 0, 2, 3, 5, 8 and 14 p.i. After excluding dead cells and gating on leukocytes (CD45^+^), four combinations of fluorochrome-labeled antibodies were used to differentiate various immune cell populations: CD11b^+^Ly6G^+^ (granulocytes, A); CD11b^+^CD115^+^ (macrophages, B); CD11c^+^MHCII^+^ (dendritic cells, C); NKp46^+^ and CD3^+^ (NK and T cells, D). The left panel illustrates the results of day 3 p.i. and indicates the population analyzed in the right panel. The right panel shows percentage of individual cell populations over time (mean±SEM). For each measurement two to three lungs were pooled and data from two independent experiments were combined (n = 3–6). Data were analyzed for statistically significant differences using non-parametric Mann-Whitney-U-test. *: p-value<0.05; **: p-value<0.01. Ratios of Ly6G^+^CD11b^high^ cells (granulocytes) vs. T cells (CD3^+^) or NK cells (NKp46^+^) in PR8M and PR8F infected mice revealed significant differences (p value as indicated) on day 5 p.i. (E).

Next, we determined the quantity and quality of cellular infiltrates of lungs in lethal (PR8F) and non-lethal (PR8M, low dose) infections on days 2, 3 and 5 in both groups and days 8 and 14 in the latter group by flow cytometry. Despite a higher number of leukocytes infiltrating the PR8F infected lungs (Figure V in File S1), we found a more pronounced myeloid response compared to PR8M infected mice at days 3 and 5 p.i. (Figure 4). A higher number of Ly6G^+^CD11b^high^ (granulocytes) and CD115^+^ cells (macrophages) were detected in PR8F compared to PR8M infected mice (Figure 4A, B). Analysis of CD11b^+^CD115^+^ cells revealed a prominent infiltration of cells from the monocyte/macrophage lineage. Expression levels of the cell surface molecules MHCII and CD11c allow distinguishing between resident macrophages (CD11c^high^MHCII^low^), exudate macrophages (CD11c^int^MHC^low^) and inflammatory dendritic cells (DCs) (CD11c^high^MHCII^high^) [24], [25]. Using these markers, we found that the relative amounts of the respective cell types differ largely between PR8F and PR8M infected mice (Figure 4C). Especially the population of highly activated inflammatory myeloid DC was much more distinctive in PR8F compared to PR8M infected mice (Figure 4C, left). On day 5 p.i., PR8M infected lungs contained a higher proportion of lymphocytes compared to lethally infected PR8F mice (Figure 4D). A strong increase of CD3^+^ cells (T cells) starting from day 5 p.i. indicated the onset of the adaptive immune response in PR8M infected animals. These T cells increased from 21% on day 3 to a maximum of 46% on day 8. On the other hand, in PR8F infected mice the innate immune cells were still dominating at day 5 p.i. and a switch to the adaptive immune cells was not detected at all; the number of CD3^+^ cells remained at a low level in PR8F infected animals.

To associate the changes in cellular infiltrates with severity of infection, we calculated different ratios of cell populations in the two infection models. The ratios of Ly6G^+^CD11b^high^ cells vs. CD3^+^ or NKp46^+^ (natural killer (NK)) cells were the best indicative parameters for a lethal infection. They were significantly higher on day 5 in the lethal compared to the non-lethal infection (Figure 4E).

In summary, the pronounced innate immune response in PR8F infected mice and the onset of the adaptive response in PR8M infected mice as observed in the lung is well reflected in the periphery.

## Discussion

Experimental animal model systems represent an extremely important tool to identify suitable markers indicating the severity of diseases. The interpretation of human studies on the host response and severe outcomes after influenza A infections are often limited by too many cofactors that cannot be controlled, such as previous vaccinations, infections, other diseases and treatments as well as nutrition, age and the general physiological condition. Another great limitation in humans is the difficult access to specimens from patients. Correlating changes in the blood with tissue damage, viral load or cellular infiltrates in the infected lung is not feasible. On the other hand, most studies that have been performed in animal models investigate in detail the primary organs affected but almost never correlate these results with changes in the peripheral blood. Therefore, we determined the changes in infected lungs and in the periphery in a well-controlled mouse model system in order to correlate peripheral markers with severity of disease, disease progression and lethal or non-lethal outcome after infection with influenza A virus.

Flow cytometric as well as histological results revealed a dominance of myeloid cells in lungs of lethally (PR8F) infected compared to non-lethal (PR8M) infected C57BL/6J mice. Our data indicate a crucial role of granulocytes in influenza pathology. This is consistent with reports investigating the excessive infiltration of innate immune cells in mice and non-human primates after infection with highly pathogenic influenza A viruses [26], [27]. It was demonstrated that the role of neutrophils varies depending on the severity of the infection, and that low dose neutrophil depletion increased survival of PR8 infected mice by reducing the damaging effects of these cells, whereas the total depletion of granulocytes caused an increase in mortality [28], [29], [30]. Here, we identified a strong accumulation of Ly6G^+^CD11b^+^ cells in the lung after lethal PR8F infections accompanying an even earlier increase of granulocytes in the blood.

The comparison of hematological and immunological analyses is limited by the fact that the definition of granulocytes is not congruent. Cells expressing CD11b and Ly6G (Gr-1) are defined as granulocytes but also as myeloid-derived suppressor cells (MDSC). The MDSC reflect a heterogeneous population of myeloid cells including progenitors and immature cells that expand during infection and inflammation with the potential to suppress T cells [31]. Furthermore, other markers like CD115 might be coexpressed defining subsets of MDSC [32]. It was demonstrated that MDSC increase upon infection with PR8 [33] and exhibit a suppressive function on the adaptive immune response [33], [34]. In line with suppressive effects associated with MDSC, we did not observe infiltration of lymphocytic immune cells into the lungs of PR8F to the same extend as in PR8M infected mice. In the blood from the latter, we detected an almost 2-fold augmentation of the lymphocyte concentration already from day 3 on. This could be interpreted as the first peripheral signs of adaptive immune cell activation and was not observed in the lethally infected mice.

In addition, analysis of the lung infiltration revealed that the antibodies used do not only reveal quantitative but also qualitative differences in the activation and maturation of *e.g.* MHCII^+^CD11c^+^ cells in the lung. C57BL/6J mice infected with PR8F as well as DBA/2J infected with PR8M (data not shown) demonstrated a higher relative amount of MHCII^+^CD11c^+^ in the lung with more cells expressing a MHCII^high^CD11c^high^ phenotype. This is indicative for a stronger inflammatory response already on day 3 p.i. [24], [25].

The investigation of hematological data revealed that non-lethally infected mice showed an initial parallel leuko- and lymphopenia followed by a leuko-/lymphocytosis, whereas lethally infected animals displayed a characteristic opposing trend of decreased lymphocytes versus increased granulocytes. We infected C57BL/6J mice with different variants of the mouse-adapted PR8 (H1N1) virus that are increasingly virulent (PR8M, PR8F, and hvPR8). A clear relation of disease severity and granulocyte to lymphocyte ratio was obtained. Most remarkably, the increase of this ratio in lethal infection scenarios was observed already within the first two days after infection whereas the same variation in the ratios of granulocytes to lymphocytes was found on day 5 p.i. in lung infiltrating immune cells. The fact that this ratio was more pronounced and occurred earlier in the periphery than in the lung supports the notion that it represents a very valuable blood indicator for influenza infection processes in the lung, at least in our experimental models. Using an additional mouse inbred strain, DBA/2J, with a known high susceptibility to influenza A infection, we verified that the characteristics of blood parameters may predict the infection process also if the genetic susceptibility of the host, and not the pathogen, is causing a severe course of the disease. With respect to individual kinetics due to the virus or the host genetics we still observed a significant correlation of the granulocyte to lymphocyte ratio and viral load. The influence of different human virus isolates on hematological parameters was investigated previously in BALB/c and C57BL/6 mice ascertaining the impact of host and pathogen determinants on the pathogenicity [15]. Applying our method to calculate the granulocyte to lymphocyte ratio for the data of this publication we found the same correlation of the ratio with disease severity. Also, in a another study [35], hematological data were determined in influenza A (pandemic and swine influenza A viruses) infected mice as well. Again we found that our method was able to relate the blood derived results with disease severity. This confirmed that our method has a general validity in a variety of murine models. When combining the results from Belser et al. [35], Otte et al. [15] and our data, we found a ratio of more than two to be critical for severe disease. A ratio of more than three always resulted in lethality. It should, however, be noted that this parameter needs to be validated in human patients before it may be used as a diagnostic tool.

Ferrets are considered as another well suitable animal model to study especially influenza transmission and pathological symptoms [36]. In this model, clinical parameters, including peripheral markers, have been investigated and correlated with disease severity [37]. Similar to our murine study, ferrets exhibited a lymphopenia during the first five days after infection. The decrease of lymphocytes, MCV (mean corpuscular volume), platelets and body weight were identified as parameters associated with mortality after infection with highly pathogenic avian influenza. As in our mouse model, the decreasing percentage of lymphocytes is accompanied by an increase of neutrophils. The change in these parameters is also depending on the virulence and serotype of the used virus. In addition, hemoglobin and hematocrit also increased and correlated with virus pathogenicity [37].

For humans, leukopenia was described to reflect severe virus infection, whereas in milder cases a lymphocytosis can be detected [38]. Clinical studies in Taiwan could associate low lymphocyte counts with respiratory failure [39]. In children, thrombocytosis occurs commonly in infections of the lower respiratory tract though an indicative correlation could not be identified [40]. In humans, the ratio of neutrophils to lymphocytes was also suggested as a diagnostic indicator for differentiating pH1N1 infections from other influenza-like illnesses [41]. Also, the lymphocyte to monocyte ratio was proposed as a blood marker for pH1N1 [42]. Thus, our results in the mouse model and studies in human patients suggest that these changes in the hemogram are valuable markers to confirm an influenza infection and distinguish it from other influenza-like illnesses. However, in order to correlate blood received results in the mouse with those from human patients, one has to consider the timing of the host response after infection. Additionally, it should be noted that an increase of blood cells might also be due to hemoconcentration during days 4 to 7 when mice demonstrate signs of severe sickness and may not eat or drink much. In our mouse model no interventions such as infusions or drug treatments were performed. This aspect has to be taken into account as well when data from mouse models are compared to results from human studies. However, cohort studies of influenza might provide in the future respective clinical results to verify if hematological data can be associated with disease severity. A parameter that can be easily obtained and correlates early with disease severity correlated with high viral loads and severe pathology of the lung, parameters that are not possible to record in humans, would represent a great improvement of present diagnostics.

In conclusion, we identified the granulocyte to lymphocyte ratio as predictive parameter for the severity of influenza A infection in mice revealing a significant correlation of this ratio with viral load in the lung already on day one after infection. This parameter was also indicative for the state and timing of the host immune response in the infected tissue. Our results might serve as a valuable basis for future investigations searching for highly predictive biomarkers in humans.

## Materials and Methods

### Ethics statement

All experiments in mice were approved according to the national guidelines of the animal welfare law in Germany. The protocol used in these experiments has been reviewed by an ethics committee and approved by the authorities (LAVES, Oldenburg, Germany; permit number: 33.9.42502-04-051/09).

### Viruses and mice

Original stocks of viruses were obtained from Stefan Ludwig, University of Münster (PR8M, A/PuertoRico/8/34 H1N1, Münster variant) and from Peter Stäheli, University of Freiburg (PR8F, A/PuertoRico/8/34 H1N1, Freiburg variant and hvPR8). Characteristics of these viruses were described previously [21]. Virus stocks were prepared by infection of 10-day-old embryonated chicken eggs as described [19]. Inbred mouse strains C57BL/6J and DBA/2J were obtained from Janvier, France and maintained under specific pathogen free conditions, according to the German animal welfare law.

### Mouse infections

Female mice (10–12 weeks of age) were anesthetized by intra-peritoneal injection with ketamin-xylazine solution (85% NaCl (0.9%), 10% ketamine, 5% xylazine; 200 µL per 20 g body weight) and then infected intra-nasally with a dose of 2×10^3^ or 2×10^5^ FFU (Focus Forming Units) in 20 µL sterile PBS. Body weight was monitored every day. Mice showing more than 30% of body weight loss were euthanized.

### Viral load in lungs

Viral load in infected lungs was determined on MDCK II (Madin-Darby Canine Kidney II) cells using the FFU assay as described [19], [21]. For determination of viral load, lungs of mice were homogenized in PBS with 0.1% BSA using the Poly Tron 2100 homogenizer. Debris was removed by centrifugation for 10 min at 1000 rpm. The samples were stored in aliquots at −70°C. Serial 10-fold dilutions of lung homogenates in DMEM containing 0.1% BSA were prepared and viral titers determined by the FFU assay.

### Histology

Lungs were prepared from mice *in toto* on indicated days after infection and immersion-fixed for 24–72 h in 4% buffered formaldehyde solution (pH 7.4), dehydrated in a series of graded alcohols and embedded in paraffin. Sections (0.5 µm) were cut with the microtome and stained with hematoxylin and eosin.

### Hematology

For monitoring hematological parameters, mice were euthanized with CO_2_ asphyxiation and blood was derived by heart puncture and collected in EDTA tubes. Blood was measured immediately in the hematologic system VetScan HM5 (Abaxis). The following parameters were analyzed: lymphocytes (Lym), granulocytes (Gr) and monocytes (Mon); red blood cells (RBC), hemoglobin (HGB), hematocrit (HCT), mean corpuscular volume (MCV), mean corpuscular hemoglobin (MCH), mean corpuscular hemoglobin concentration (MCHC), and red blood cell distribution width (RDWc); platelets (PLT), plateletcrit (PCT), mean platelet volume (MPV), and platelet distribution width (PDWc). To determine the ratio of granulocytes to lymphocytes in the blood, we used the absolute cell counts for each population, calculated their ratio and normalized this value to the value in non-infected control animals.

### Arterial oxygen saturation

Arterial oxygen saturation of infected mice was monitored by using the pulse oxymeter MouseOx (Starr life sciences corp.). The sensor clip was fixed in the neck of a single mouse which was anesthetized with isoflurane in a narcosis chamber (2% of isoflurane in pure oxygen) before measurements. When constant signals were reached, recording was stopped and data were saved digitally.

### Flow cytometry

Immune cell populations were analyzed in cell suspensions derived from homogenized lungs or whole blood. Blood was collected as described for the hematological investigations. To prevent unspecific binding to Fc receptors, antibody against CD16/32 (clone 2.4G2) was added. Cells were directly stained in two different panels with the following antibodies: αCD4-PE (BD Bioscience, Europe), αCD8-APC, αCD11b-APC, αCD19-FITC, αCD115-Alexa Fluor 488, αLy-6G/Ly-6C-PE and αNKp46-PerCP-eFluor 710 (eBioscience). After incubation (4°C, 30 min, in the dark), erythrocytes were lysed and cells fixed (FACS Lysing Solution, BD Bioscience), and washed twice with PBS/2%FCS. Lungs were isolated and passed through a 40 µm-cell strainer. Two to three homogenized lungs were pooled and a density centrifugation using Lympholyte M (Cedarlane) was performed. After washing the cells twice with PBS/2%FCS, Fc receptors were blocked with αCD16/32 and subsequently stained with αCD45-APC (eBioscience) or αCD45-FITC (ImmunoTools, Friesoythe, Germany). In addition to the antibodies described above, we used: αCD3ε-FITC, αCD11b-PE (ImmunoTools, Friesoythe, Germany), αCD11c-PE and αMHCII-FITC (eBioscience). To exclude dead cells, propidium iodide was added to each sample. Samples were measured using an Accuri C6 flow cytometer (BD Bioscience) and data were analyzed using FlowJo version 7.6.5 (Tree Star, Ashland, Oregon).

### Statistics

Data were analyzed using GraphPad Prism version 5.04 for Windows (GraphPad Software, San Diego, California). Mean±SEM were calculated for all groups. The non-parametric Mann-Whitney-U-test was used to determine p-values for the significance of differences between groups. P-values of ≤0.05 were considered significant.

## Supporting Information

File S1 Figures: Fig. I: The phenotypes of PR8 infected C57BL/6J (B6) and DBA/2J (D2) mice (Fig. IA) and histology of PR8M vs. PR8F infected B6 (Fig. IB) are displayed. Fig. II: Flow cytometric analyses of blood lymphocytes of PR8M and PR8F infected B6 are depicted. Fig. III+IV: Graphic representation of additional hematological red blood cell (Fig. III) and platelet related (Fig. IV) parameters determined from PR8M and PR8F infected B6 mice are provided. Fig. V: The absolute numbers of leucocytes in the lungs of PR8M and PR8F infected B6 mice were determined by flow cytometry. Tables: All hematological parameters determined are listed in Table I: B6 mice infected with 2×10^3^FFU PR8M; Table II: B6 mice infected with 2×10^5^FFU PR8M; Table III: B6 mice infected with 2×10^3^FFU PR8F; Table IV: B6 mice infected with 2×10^3^FFU hvPR8; Table V: D2 mice infected with 2×10^3^FFU PR8M.
(PDF)
