# Supplementary material for: Cellular Changes in Blood Indicate Severe Respiratory Disease during Influenza Infections in Mice
Source: PLoS One. 2014 Jul 24;9(7):e103149. doi: 10.1371/journal.pone.0103149 (PMC4110021; doi:10.1371/journal.pone.0103149)
Supplement: File S1 — Figures: Fig. I: The phenotypes of PR8 infected C57BL/6J (B6) and DBA/2J (D2) mice (Fig. IA) and histology of PR8M vs. PR8F infected B6 (Fig. IB) are displayed. Fig. II: Flow cytometric analyses of blood lymphocytes of PR8M and PR8F infected B6 are depicted. Fig. III+IV: Graphic representation of additional hematological red blood cell (Fig. III) and platelet related (Fig. IV) parameters determined from PR8M and PR8F infected B6 mice are provided. Fig. V: The absolute numbers of leucocytes in the lungs of PR8M and PR8F infected B6 mice were determined by flow cytometry. Tables: All hematological parameters determined are listed in Table I: B6 mice infected with 2×10^3^FFU PR8M; Table II: B6 mice infected with 2×10^5^FFU PR8M; Table III: B6 mice infected with 2×10^3^FFU PR8F; Table IV: B6 mice infected with 2×10^3^FFU hvPR8; Table V: D2 mice infected with 2×10^3^FFU PR8M. (PDF) [file pone.0103149.s001.pdf]

Supporting information: Figure I: general phenotypes

Female C57BL/6J & DBA/2J mice were infected intra-nasally with 2x10^3 FFU. Body weight loss (n=9), virus titer (n=3) and oxygen saturation (n=9) were monitored until day 14 p.i. in surviving animals (a: B6 - PR8M) or until death (b: B6 - PR8F & c: D2 - PR8M) (A). Representative histological sections of lungs (B) from non-infected controls (a) and mice infected with PR8M (b-g; days (d) 2, 4, 5, 8, 10, 14) or PR8F (h-j; d 2, 4) were prepared and stained with HE (n=3).

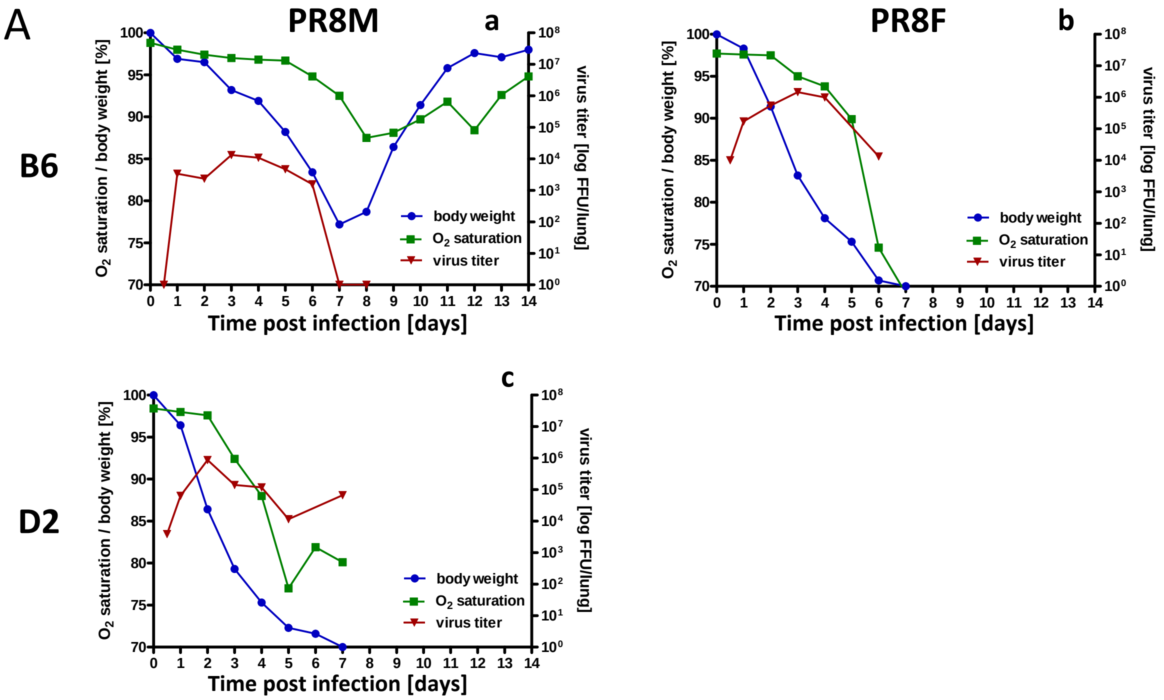

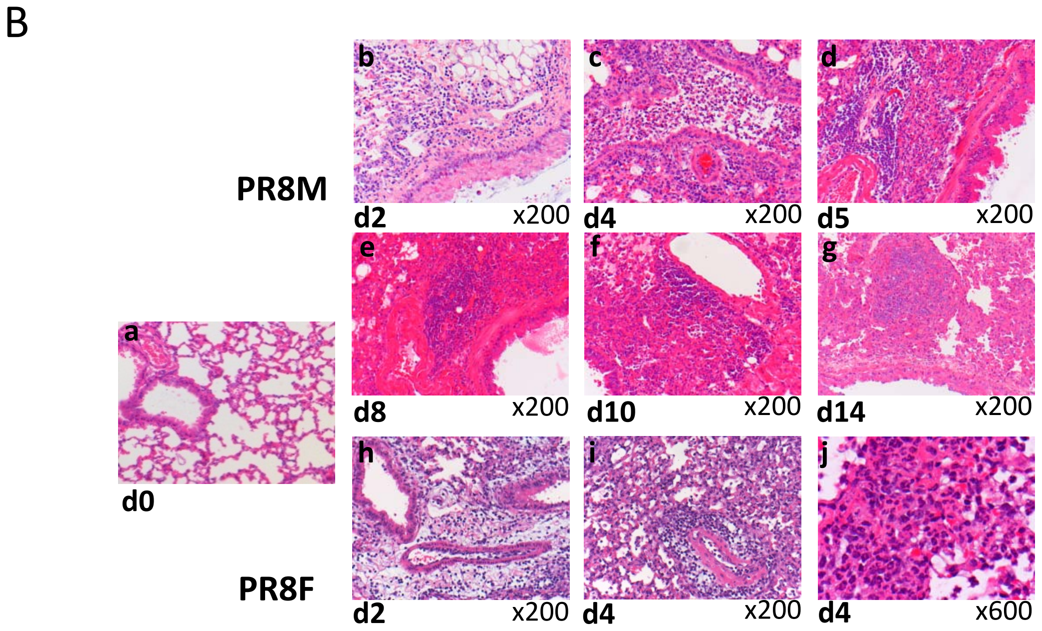

Supporting information: Figure II: Flow cytometry of blood

Lymphocytes from infected C57BL/6J mice (2x10^{3}FFU) were differentiated by flow cytometry with respect to the expression of various cell surface markers: CD19 (B cells), NKp46 (NK cells), CD4 (T helper cells) and CD8 (cytotoxic T cells). The percentage of the individual cell populations gated in relation to all lymphocytes is depicted for days 1 to 14 (PR8M) or for days 1 to 5 (PR8F) p.i., respectively. Mean±SEM values were calculated from two independent experiments (n=6). Data were analyzed for statistically significant differences using non-parametric Mann-Whitney-U-test. *: p-value<0.05; **: p-value<0.01.

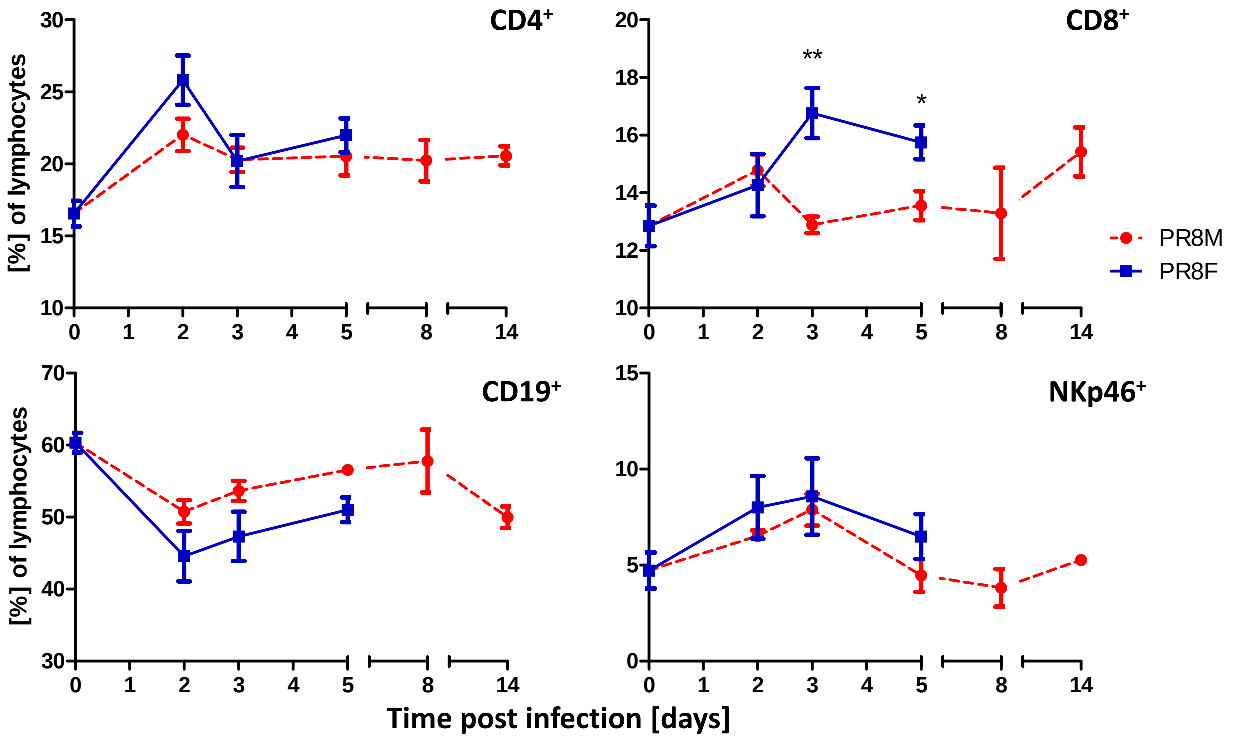

Supporting information: Figure III: red blood cell parameters

Several hematological parameters were determined from PR8M (red) and PR8F (blue) infected C57BL/6J mice (2x10^3 FFU) on days 1 to 5 p.i.: red blood cells (RBC), hemoglobin (HGB), hematocrit (HCT), mean corpuscular volume (MCV), mean corpuscular hemoglobin (MCH), mean corpuscular hemoglobin concentration (MCHC), and red blood cell distribution width (RDWc). Data were collected from two independent experiments (total n=10) for the first five days p.i. Data were analyzed for statistically significant differences using non-parametric Mann-Whitney-U-test. *: p value<0.05; **: p value<0.01; ***: p value<0.001.

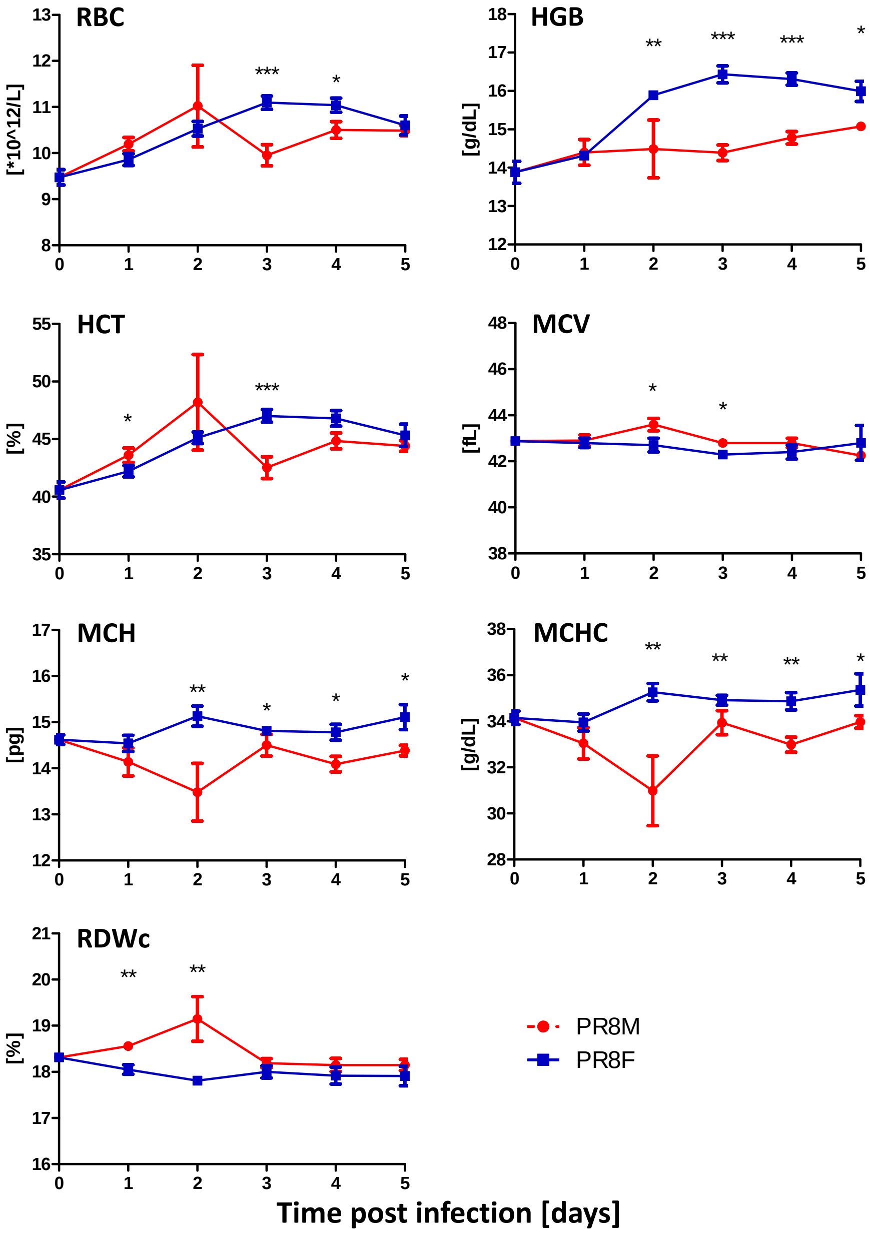

Supporting information: Figure IV: platelet parameters

Various platelet associated parameters were determined in the blood of PR8M (red) and PR8F (blue) infected C57BL/6J mice ($2x10^3$FFU) during the course of infection: platelets (PLT), plateletcrit (PCT), mean platelet volume (MPV), and platelet distribution width (PDWc). Data were collected from two independent experiments (total n=10) for the first five days p.i. Data were analyzed for statistically significant differences using non-parametric Mann-Whitney-U-test. *: p value<0.05; **: p-value<0.01.

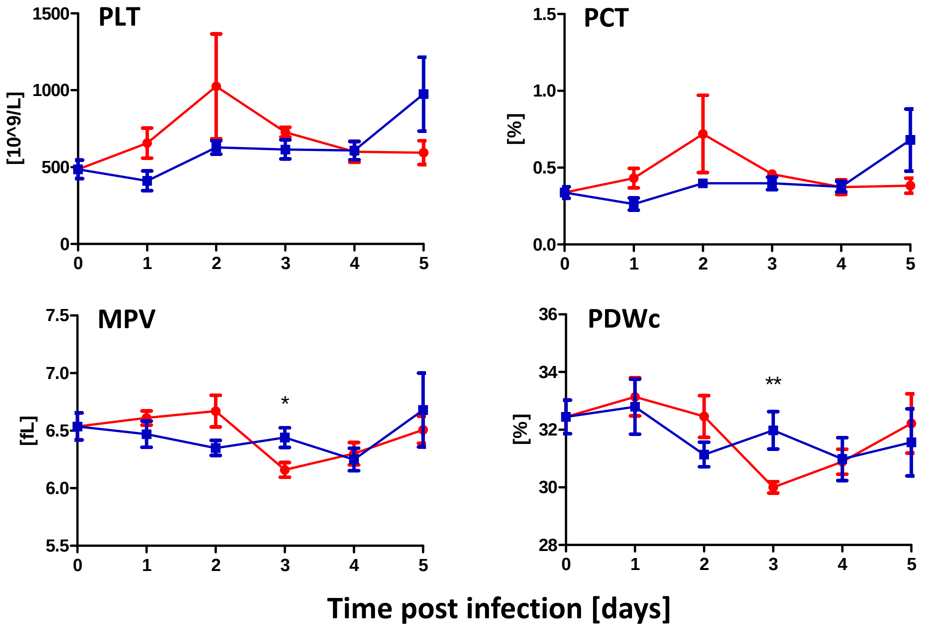

Supporting information: Figure V: Flow cytometry – absolute leukocyte counts

Flow cytometric analyses revealed quantitative differences of immune cell infiltration after PR8M versus PR8F infection. Lungs from infected C57BL/6J mice (2x10^3FFU) were extracted and cell suspensions were analyzed by flow cytometry on days 0, 2, 3, 5, 8 and 14 p.i. After excluding dead cells leukocytes ($CD45^+$) were gated. For each measurement two to three lungs were pooled and data from two independent experiments were combined (n=12-26). Data were analyzed for statistically significant differences using non-parametric Mann-Whitney-U-test. *: p-value<0.05; ****: p-value<0.0001.

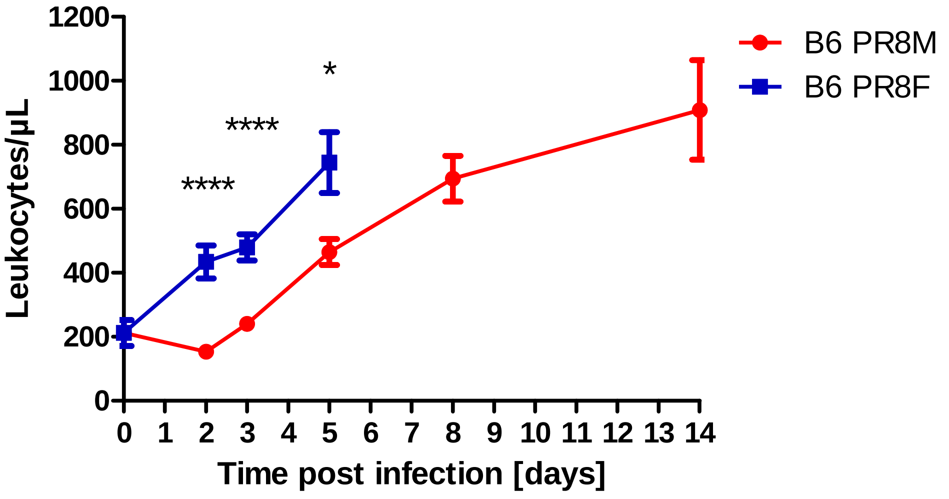

Supporting information: Table I – C57BL/6J mice infected with PR8M, $2x10^3$FFU

**B6 PR8M2x10^3**																						
day post infection	0		1		2		3		4		5		6		8		10		12		14	
n	33		10		10		10		10		12		5		5		5		5		5	
	Mean	SEM	Mean	SEM	Mean	SEM	Mean	SEM	Mean	SEM	Mean	SEM	Mean	SEM	Mean	SEM	Mean	SEM	Mean	SEM	Mean	SEM
**WBC [10^9/L]**	12.31	0.96	7.15	0.54	7.73	1.09	6.83	0.49	10.04	1.54	13.53	1.40	18.89	1.50	21.89	1.02	16.73	1.11	16.29	0.81	15.45	2.08
**Lym [10^9/L]**	9.66	0.66	6.31	0.49	6.23	0.97	5.46	0.39	7.54	0.97	9.99	0.79	13.25	1.55	16.85	1.03	13.02	1.16	13.26	0.80	11.29	1.74
**Mon [10^9/L]**	0.36	0.08	0.15	0.04	0.27	0.08	0.17	0.04	0.40	0.15	0.49	0.16	0.99	0.41	0.13	0.00	0.34	0.24	0.46	0.25	1.11	0.44
**Neu [10^9/L]**	2.48	0.36	0.69	0.10	1.22	0.19	1.21	0.12	2.10	0.54	3.05	0.66	4.64	0.50	4.32	0.61	3.37	0.26	2.57	0.36	3.06	0.40
**Lym [%]**	80.92	1.41	88.20	1.24	79.37	2.02	80.01	1.39	78.00	2.64	75.80	2.15	69.16	3.82	76.74	1.07	77.36	1.90	81.28	1.29	71.86	2.76
**Mon [%]**	2.61	0.46	2.27	0.55	3.67	0.90	2.31	0.45	3.21	0.74	3.33	0.83	4.84	1.85	0.60	0.00	2.24	1.64	2.84	1.52	6.24	2.30
**Neu [%]**	16.47	1.38	9.54	1.02	16.95	2.02	17.68	1.20	18.78	2.44	20.87	2.23	26.00	5.12	22.66	1.07	20.46	2.03	15.88	2.25	21.86	4.86
**RBC [*10^12/L]**	9.47	0.17	10.20	0.15	11.02	0.88	9.95	0.23	10.50	0.18	10.49	0.11	9.65	0.43	10.71	0.12	11.27	0.98	9.65	0.10	10.08	0.33
**HGB [g/dL]**	13.88	0.28	14.40	0.33	14.49	0.75	14.39	0.20	14.78	0.16	15.08	0.11	15.04	0.20	16.36	0.17	15.56	0.21	14.70	0.11	15.28	0.25
**HCT [%]**	40.58	0.70	43.61	0.62	48.20	4.15	42.52	0.95	44.85	0.68	44.40	0.47	40.57	1.79	45.43	0.57	42.39	0.77	39.68	0.55	41.60	1.17
**MCV [fL]**	42.88	0.16	42.90	0.23	43.60	0.27	42.80	0.13	42.80	0.20	42.25	0.13	42.20	0.20	42.20	0.20	41.20	0.37	41.00	0.32	41.40	0.24
**MCH [pg]**	14.62	0.10	14.14	0.30	13.48	0.63	14.50	0.23	14.09	0.17	14.38	0.11	15.76	0.94	15.30	0.04	15.14	0.17	15.22	0.08	15.16	0.32
**MCHC [g/dL]**	34.15	0.29	33.05	0.68	30.99	1.52	33.94	0.52	32.99	0.32	33.98	0.27	37.50	2.26	36.00	0.12	36.68	0.40	37.06	0.42	36.74	0.63
**RDWc**	18.32	0.08	18.56	0.09	19.15	0.48	18.19	0.10	18.15	0.14	18.15	0.12	18.32	0.19	21.24	2.70	18.64	0.10	19.30	0.51	19.00	0.15
**PLT**	511.32	54.33	656.30	98.67	1025.20	341.42	727.10	32.03	601.10	68.62	594.25	77.16	343.60	49.11	378.00	44.71	284.60	27.91	199.20	9.32	214.80	28.53
**PCT**	0.33	0.04	0.43	0.07	0.71	0.25	0.45	0.02	0.38	0.04	0.38	0.05	0.27	0.05	0.31	0.04	0.22	0.03	0.15	0.01	0.16	0.03
**MPV**	6.54	0.12	6.61	0.06	6.67	0.14	6.16	0.06	6.30	0.10	6.51	0.12	7.66	0.27	8.10	0.35	7.42	0.32	7.52	0.18	7.50	0.32
**PDWc**	32.44	0.58	33.14	0.66	32.46	0.72	30.00	0.20	30.89	0.43	32.22	1.03	37.04	0.70	37.26	0.90	35.42	0.82	35.40	0.37	34.44	1.31

Supporting information: Table II- C57BL/6J mice infected with PR8M, 2x10^5FFU

**B6 PR8M 2x10^5**												
day post infection	0		1		2		3		4		5	
n	33		10		10		10		10		10	
	Mean	SEM	Mean	SEM	Mean	SEM	Mean	SEM	Mean	SEM	Mean	SEM
**WBC [10^9/L]**	12.31	0.96	12.25	1.35	9.85	1.20	14.20	1.00	16.35	1.95	16.22	1.52
**Lym [10^9/L]**	9.66	0.66	8.70	0.89	6.33	0.65	8.37	0.66	11.33	1.28	10.68	0.96
**Mon [10^9/L]**	0.36	0.08	0.45	0.13	0.44	0.14	1.00	0.19	0.86	0.21	0.77	0.24
**Neu [10^9/L]**	2.48	0.36	3.09	0.46	3.05	0.54	4.79	0.34	4.16	0.57	4.77	0.66
**Lym [%]**	80.92	1.41	71.61	1.53	66.35	2.97	59.11	1.78	70.16	1.34	66.41	2.02
**Mon [%]**	2.61	0.46	3.85	0.86	3.82	0.82	6.80	1.19	4.97	0.81	4.93	1.37
**Neu [%]**	16.47	1.38	24.53	1.56	29.80	2.68	34.08	1.55	24.82	1.33	28.73	2.14
**RBC [*10^12/L]**	9.47	0.17	9.58	0.27	10.52	0.23	10.78	0.16	10.72	0.09	9.96	0.26
**HGB [g/dL]**	13.88	0.28	14.55	0.11	16.14	0.16	16.28	0.09	15.56	0.16	15.35	0.15
**HCT [%]**	40.58	0.70	40.86	1.20	44.58	1.07	45.59	0.72	45.37	0.41	42.48	1.06
**MCV [fL]**	42.88	0.16	42.70	0.21	42.30	0.26	42.30	0.21	42.20	0.29	42.70	0.30
**MCH [pg]**	14.62	0.10	15.35	0.57	15.39	0.32	15.13	0.26	14.53	0.14	15.51	0.46
**MCHC [g/dL]**	34.15	0.29	35.96	1.39	36.35	0.86	35.79	0.65	34.32	0.33	36.37	1.11
**RDWc [%]**	18.32	0.08	18.11	0.07	18.34	0.32	18.08	0.22	18.04	0.14	17.64	0.18
**PLT [10^9/L]**	511.32	54.33	428.30	50.46	718.30	75.15	570.60	68.51	618.70	61.47	618.8	99.18
**PCT [%]**	0.33	0.04	0.28	0.03	0.46	0.05	0.37	0.05	0.38	0.04	0.38	0.06
**MPV [fL]**	6.54	0.12	6.55	0.20	6.36	0.06	6.44	0.13	6.23	0.10	6.16	0.11
**PDWc [%]**	32.44	0.58	33.13	1.02	32.37	0.74	32.34	0.70	31.62	0.82	29.94	1.18

Supporting information: Table III- C57BL/6J mice infected with PR8F,2x10^3FFU

**B6 PR8F2x10^3**												
day post infection	0		1		2		3		4		5	
n	33		10		10		10		10		12	
	Mean	SEM	Mean	SEM	Mean	SEM	Mean	SEM	Mean	SEM	Mean	SEM
**WBC [10^9/L]**	12.31	0.96	13.39	1.52	10.27	0.74	14.00	1.06	11.33	1.79	14.26	1.70
**Lym [10^9/L]**	9.66	0.66	10.18	1.07	7.24	0.53	7.01	0.67	6.13	1.18	8.70	1.11
**Mon [10^9/L]**	0.36	0.08	0.39	0.16	0.47	0.09	0.94	0.22	0.76	0.22	1.08	0.23
**Neu [10^9/L]**	2.48	0.36	2.82	0.59	2.56	0.28	5.75	0.52	4.45	0.51	4.48	0.68
**Lym [%]**	80.92	1.41	77.01	2.37	70.64	1.62	53.13	2.16	52.21	1.98	60.49	3.20
**Mon [%]**	2.61	0.46	3.13	1.20	4.58	0.82	6.07	1.17	5.70	1.03	7.39	1.18
**Neu [%]**	16.47	1.38	19.86	3.01	24.79	1.91	40.80	1.75	42.10	2.66	32.11	3.47
**RBC [*10^12/L]**	9.47	0.17	9.86	0.13	10.53	0.16	11.10	0.14	11.04	0.15	10.60	0.20
**HGB [g/dL]**	13.88	0.28	14.32	0.07	15.89	0.08	16.43	0.22	16.31	0.16	15.99	0.26
**HCT [%]**	40.58	0.70	42.21	0.49	45.11	0.50	47.02	0.54	46.80	0.67	45.32	0.97
**MCV [fL]**	42.88	0.16	42.80	0.20	42.70	0.30	42.30	0.15	42.40	0.31	42.80	0.76
**MCH [pg]**	14.62	0.10	14.54	0.17	15.13	0.22	14.81	0.09	14.78	0.17	15.11	0.27
**MCHC [g/dL]**	34.15	0.29	33.95	0.37	35.26	0.38	34.92	0.21	34.87	0.38	35.36	0.70
**RDWc**	18.32	0.08	18.05	0.10	17.81	0.08	18.00	0.13	17.92	0.18	17.91	0.21
**PLT**	511.32	54.33	411.10	64.69	628.00	44.20	614.90	62.29	607.80	59.24	975.00	241.15
**PCT**	0.33	0.04	0.26	0.04	0.40	0.03	0.40	0.04	0.38	0.03	0.68	0.20
**MPV**	6.54	0.12	6.47	0.11	6.35	0.07	6.44	0.09	6.25	0.10	6.68	0.32
**PDWc**	32.44	0.58	32.80	0.96	31.14	0.43	31.98	0.65	30.98	0.74	31.56	1.16

Supporting information: Table IV-C57BL/6J mice infected with hvPR8, $2x10^3$FFU

**B6 hvPR8 2x10^3**												
day post infection	0		1		2		3		4		5	
n	33		10		10		10		10		10	
	Mean	SEM	Mean	SEM	Mean	SEM	Mean	SEM	Mean	SEM	Mean	SEM
**WBC [10^9/L]**	12,31	0,96	15,50	1,19	9,90	1,13	14,51	1,67	12,78	1,97	11,36	2,24
**Lym [10^9/L]**	9,66	0,66	10,98	0,73	5,74	0,72	7,46	1,03	6,66	1,17	6,12	1,23
**Mon [10^9/L]**	0,36	0,08	1,05	0,17	0,50	0,10	1,62	0,76	1,12	0,24	1,13	0,27
**Neu [10^9/L]**	2,48	0,36	3,47	0,51	3,67	0,44	6,15	0,55	5,00	0,64	4,11	0,82
**Lym [%]**	80,92	1,41	71,59	1,99	57,44	2,16	51,04	1,87	50,56	2,65	53,74	3,30
**Mon [%]**	2,61	0,46	6,84	1,08	5,20	0,89	5,47	1,22	8,37	0,95	8,93	0,65
**Neu [%]**	16,47	1,38	21,57	2,44	37,37	2,31	43,50	2,18	41,08	2,48	37,35	3,29
**RBC [*10^12/L]**	9,47	0,17	9,92	0,13	10,75	0,12	11,11	0,10	11,11	0,21	11,64	0,24
**HGB [g/dL]**	13,88	0,28	14,69	0,12	15,83	0,21	16,36	0,14	16,62	0,13	17,44	0,22
**HCT [%]**	40,58	0,70	42,58	0,54	45,82	0,55	47,16	0,41	47,02	0,94	50,11	0,93
**MCV [fL]**	42,88	0,16	43,10	0,10	42,60	0,16	42,40	0,16	42,50	0,34	43,22	0,97
**MCH [pg]**	14,62	0,10	14,81	0,16	14,73	0,16	14,73	0,14	15,03	0,28	15,03	0,26
**MCHC [g/dL]**	34,15	0,29	34,49	0,37	34,58	0,44	34,72	0,38	35,48	0,66	34,87	0,44
**RDWc**	18,32	0,08	18,04	0,11	18,00	0,13	18,03	0,12	17,94	0,24	18,26	0,15
**PLT**	511,32	54,33	435,90	49,60	642,90	81,85	656,20	70,10	786,90	49,68	1015,65	334,18
**PCT**	0,33	0,04	0,28	0,03	0,41	0,05	0,42	0,05	0,52	0,03	0,93	0,19
**MPV**	6,54	0,12	6,61	0,17	6,42	0,12	6,37	0,14	6,70	0,16	6,84	0,30
**PDWc**	32,44	0,58	32,04	0,86	32,36	0,83	31,89	0,76	31,75	0,92	33,26	0,69

Supporting information: Table V- DBA/2J mice infected with PR8M, 2x10^{3}FFU

**D2 PR8M2x10^3**												
day post infection	0		1		2		3		4		5	
n	24-29		10		9		10		10		12	
	Mean	SEM	Mean	SEM	Mean	SEM	Mean	SEM	Mean	SEM	Mean	SEM
**WBC [10^9/L]**	16.15	0.77	8.25	0.59	7.72	0.88	7.35	0.25	11.85	1.87	15.48	1.75
**Lym [10^9/L]**	11.15	0.53	6.73	0.42	4.38	0.62	3.82	0.21	6.75	1.12	8.99	1.12
**Mon [10^9/L]**	0.51	0.12	0.21	0.03	0.33	0.05	0.41	0.07	0.52	0.14	0.69	0.18
**Neu [10^9/L]**	4.50	0.29	1.29	0.18	3.02	0.31	3.13	0.18	4.57	0.78	5.80	0.76
**Lym [%]**	69.39	1.14	82.40	1.26	55.41	2.91	51.87	2.22	57.13	2.10	57.99	3.25
**Mon [%]**	3.05	0.68	2.62	0.38	4.24	0.61	5.55	0.82	4.88	0.96	4.46	0.88
**Neu [%]**	27.56	1.25	14.98	1.32	40.33	2.64	42.61	2.16	37.99	1.46	37.54	3.46
**RBC [*10^12/L]**	10.89	0.13	11.11	0.18	10.34	0.68	11.93	0.12	12.11	0.20	12.87	0.20
**HGB [g/dL]**	14.70	0.06	14.53	0.23	13.13	0.85	15.64	0.13	15.99	0.20	16.95	0.34
**HCT [%]**	45.48	0.54	47.12	0.70	44.37	2.76	49.78	0.48	50.00	0.71	52.42	0.82
**MCV [fL]**	41.75	0.25	42.30	0.33	42.89	0.45	41.80	0.25	41.30	0.26	40.70	0.21
**MCH [pg]**	13.55	0.17	13.08	0.12	12.74	0.15	13.10	0.14	13.21	0.16	13.16	0.07
**MCHC [g/dL]**	32.44	0.42	30.84	0.33	29.61	0.39	31.41	0.30	32.02	0.34	32.34	0.27
**RDWc**	19.88	0.15	20.38	0.12	20.52	0.23	20.04	0.22	19.95	0.44	19.77	0.29
**PLT**	342.79	59.22	683.20	103.20	680.78	112.85	937.20	68.67	692.30	126.79	706.10	136.73
**PCT**	0.23	0.04	0.47	0.07	0.49	0.09	0.66	0.05	0.48	0.09	0.51	0.10
**MPV**	6.68	0.11	6.76	0.15	7.11	0.14	6.97	0.19	6.76	0.20	7.09	0.18
**PDWc**	34.01	0.47	34.33	0.50	35.56	0.74	34.65	0.97	34.84	0.98	36.61	0.90
